# Tropical Abscess of the Liver

**Published:** 1904-03

**Authors:** J. E. Thompson

**Affiliations:** Galveston, Texas


### TEXAS MEDICAL JOURNAL.

ESTABLISHED JULY, 1885.

PUBLISHED MONTHLY.—SUBSCRIPTION $1.00 A YEAR.

VOL. XIX. AUSTIN, MARCH, 1904. No. 9.

For Texas Medical Journal.

### Tropical Abscess of the Liver.

PROFESSOR J. E. THOMPSON, M. D., GALVESTON, TEXAS.

During the past ten years my experience with tropical abscess of the liver has been sufficiently extensive to lead me to think that a discussion of the most interesting phases of this affection would be instructive.

As to the etiology, in a large portion of cases there was no history of dysentery; but in others no history could be obtained, and, further, a careful examination of the stools failed to reveal any trace of amœbæ. In two cases of amœbic abscess that ended fatally the autopsy revealed no evidence of ulceration either in the colon or the small intestine. In the rest of the autopsies the colon showed lesions, in some extensive recent ulcerations, in others ulcerated patches and healed scars. Dysentery present in between 50 to 60 per cent. of all cases. McLeod found it in thirty-nine out of forty cases of hepatic abscess.

The bacteriological examination of the pus varied greatly. Many of the abscess cavities were absolutely sterile, as shown by cultivations made in agar, blood serum, and bouillon, taken at ordinary temperatures and in the presence of air. In one case an anaerobic bacillus was found very similar in appearance to the "bacillus anaerobicus capsulatus of Welch." This sterility as regards the ordinary pyogenic organisms is interesting, and probably explains the absence of serious symptoms after aspirating with large needles. The escape of a small quantity of pus from such a puncture of the liver can hardly be avoided, particularly when the abscess is near the surface, and if the pus were infected peritonitis would probably result. The sterility of these cavities as regards putrefactive and other organism also points to the necessity for great care in treatment. I have seen operations on large hepatic abscesses, which were conducted with the best aseptic surroundings, followed by a normal temperature for one or two weeks. Afterwards, in spite of most efficient drainage, the temperature would rise and become hectic in its type, owing to infection spreading along the track of the drainage tube. Examination would then show the pus full of both putrefactive and pyogenic organisms.

The *symptoms* of hepatic abscess are sometimes so definite that a diagnosis is often made in a few minutes. Such a group of symptoms as the following would be absolutely conclusive of a positive diagnosis: "A history of dysentery, general feeling of malaise, elevated temperature, yellow sallow appearance of the skin, at times approaching to jaundice, pain in the hepatic region or over the shoulder blade, or at the point of acromion, or in all three places, enlargement of the liver either upwards or downwards, or in both directions, tenderness over the hepatic region, bulging of the intercostal spaces, œdema over the lower costal zone, and irritating spasmodic cough.

It is rarely that one finds all these symptoms present together in the same case, but in quite a considerable number of cases that are in the advanced stages the greater number of the above quoted symptoms will be present. It will be well to discuss these symptoms seriatim.

*History of dysentery.* In a large proportion of cases this is found, but, in quite a considerable number, as previously stated, no evidence, either ante or post mortem, can be discovered. Great stress must be laid on a positive finding of mucus and blood in the stools, especially if amœbæ are present in the mucus, but a negative finding is of little value.

*General feeling of malaise.* This is often present weeks and months before definite positive symptoms show themselves. If the patient is under careful observation during this period, a febrile temperature curve will almost always be found. A careful blood examination will show a slight degree of leucocytosis, diminution of red blood corpuscles, and decrease in quantity of hemoglobin.

It is during this period that the patient begins to show the *yellow sallow appearance* due to anemia, and in malarial districts the cases are almost invariably diagnosed as cases of malaria. True jaundice, however, occurs in a few cases, and is rarely very intense. A careful blood examination for the malarial parasite would enable this mistake to be avoided. Quite a number of cases have come under my care from the malarial districts of the State, with a clear history of two or three months treatment for malaria, where no suspicion of an abscess of the liver ever crossed the mind of the attendant. Manson states that it is probably present in from 50 to 60 per cent. of all cases. McLeod found it present in thirty-nine out of forty cases of hepatic abscess, and as this one recovered, it might have been present in this.

*Elevated temperature.* This is probably the most deceptive symptom. In some cases at the inception of the disease the temperature curve is irregular. It is rarely higher than 101 degrees, and for days at times it may fail to reach a higher point than 99 or 99.5 degrees. It is rarely absent in the evenings. In some cases the febrile symptoms seem to be absent for some days, returning later in paroxysms resembling malaria. It is very probable that careful examination will demonstrate that cases, associated with afebrile intervals, are much rarer than is supposed. Personally, I have never seen an afebrile case. The most usual curve is remittent, the temperature varying between 101 and 103 degrees in the evening and 99 and 100 degrees in the morning.

The cause for the fever in sterile abscess must be sought for partly in altered metabolism and partly in absorption of degenerated liver tissue.

*Pain.* This is best considered in three distinct situations.

1. *In the region of the liver.* In the majority of cases pain is present in this situation during the course of the disease. Sometimes it may be the prominent symptom and may be constantly present in the initial stages, but later it may give place to pain in the other situations. It is usually of a dull aching character, and is constantly present. In the later stages it may be of a sharp stabbing nature, and is produced by a localized inflammation of the peritoneal surface of the liver over the pointing abscess. Tenderness over the hepatic region is usually not very pronounced, but in some few cases it may be very marked. I have seen one case where moderate pressure on all the intercostal spaces over the right lobe of the liver produced intense pain.

2. *Over the shoulder blade* pain is also common. It is in its character very similar to the pain produced by simple hepatic congestion, that is common in individuals who are the subjects of so-called "sluggish liver." One of my latest cases, with an abscess in the right lobe, showed this symptom on the right side, unassociated with any pain over the hepatic region.

3. *At the point of the acromion.* This symptom when present is very characteristic. It is situated at the very point of the shoulder, and is of a dull agonizing nature. I have found it present on the right side in abscess of the right lobe and vice versa. I have also found this symptom very marked in a case of peri-hepatic abscess following appendicitis. In this case the abscess first formed around the right lobe and pain was then felt at the tip of the right shoulder. Later it developed around the left lobe, and then pain was pronounced at the point of the left shoulder, and was the means of enabling us to diagnose the position of the pus focus.

In this connection I remember allowing this symptom to slip me in one of my earliest cases. The patient, a man of 45, came under my care with the following symptoms: Fever of a remittent type, epigastric tenderness, constant vomiting, progressive emaciation, and marked pain at the point of the left shoulder. The symptoms had been present about four months, and had gradually increased in severity. I did not suspect hepatic abscess, but rather favored the idea that we had to deal with a carcinoma of the stomach; particularly as an examination of the gastric contents showed an absence of hydrochloric acid. An exploratory operation was decided upon. The stomach was found healthy, and there was no evidence of disease in either right or left lobe of the liver, although I must admit that my examination of the left lobe was not as complete as it ought to have been. The patient lived four days only. During his time, from careful analysis of the symptoms, and owing chiefly to the severity of the pain in the tip of the left shoulder, I became imbued with the idea that I had overlooked an abscess in the left lobe. I obtained an autopsy and found an abscess cavity in the left lobe as large as a tangerine orange.

Since that time I have found the symptom invaluable. When present and accompanied by febrile symptoms and signs of hepatic enlargement it is quite conclusive.

*Enlargement of the liver.* This may occur either upwards or downwards or in both directions. Slight enlargements upwards are very difficult to detect. Marked enlargements temporarily obliterate the lower portion of the pleural space and push the lower edge of the lung upwards. It is usually easy to diagnose between the pushing of the diaphragm upwards by an enlarged liver, and a collection of fluid in the pleural cavity. If the patient lies on the left side the liver and diaphragm will drop away from the affected side and the lung will follow to a lower level. In cases of effusion into the pleural cavity, the level of the dullness will remain fixed. Contrary to what is usually believed in marked enlargement of the right lobe of the liver upwards "Litten's sign" is absent. Enlargement downwards is usually easy of diagnosis, but where the enlargement occupies the posterior edge of the liver, it may become marked before it is detected. One of my cases with marked ascites (the only one I have met with ascitic fluid in the abdominal cavity), showed most of the symptoms except hepatic enlargement. After evacuation of the ascitic fluid, I found the under surface of the liver roughened and tuberculated. There was a marked bulging of the posterior part of the lower surface. I found this corresponded to the Spigelian lobe and the adjacent part of the liver. An opening was made through the gastro-hepatic omentum, and, after careful gauzing, the abscess was evacuated.

*Bulging of the intercostal spaces* is a very valuable sign if associated with other symptoms. Its cause must be carefully analyzed, for it may be caused by pleural effusions. If due to hepatic abscess, the lower portion of the pleural cavity is invariably obliterated and the pus is quite near the surface.

*Œdema over the lower costal zone* is probably one of the most valuable signs for localization. It is almost invariably placed over the most superficial part of the abscess cavity. It is the place we always choose for the insertion of the exploring needle. The spots of œdema are not large as a rule. They are about the size of a penny.

*Irritating Spasmodic Cough.*—This is only of value if associated with other symptoms. It is usually evidence of diaphragmatic involvement, although I have seen it marked in cases where the diaphragm was intact. In cases where the abscess is passing through the diaphragm into pleural cavity or into adherent lung, it is a prominent symptom, and precedes by weeks or months the final bursting of the abscess into a bronchus.

I have seen the same sign in a "left pyo nephrosis," due to renal calculi; and this possibility must always be considered in making a differential diagnosis.

Ocular demonstration of hepatic pus must, however, be the chief link in the evidence. Until we actually see the pus we can not definitely say that we have a hepatic abscess; and no operation should be attempted until the position of the abscess cavity is definitely fixed. Of course, a careful blood examination is of great value. Leucocytosis is invariably present, and the iodine reaction is very reliable.

The method of aspiration is very important. If carelessly conducted, or if a defective aspirator is used, failure may attend the effort, even though the abscess cavity be penetrated. A perfect syringe and a needle must be used. The moment the needle penetrates the skin a vacuum must be made, and the needle slowly pushed on. It is usually bad practice to use a long piece of rubber tubing between the needle and the barrel of the syringe, because, in small abscesses, the pus may not be sufficient in quantity to fill up the tubing, and so may escape detection until the needle is withdrawn. Slow penetration is necessary, because, if we pass too rapidly across a small abscess cavity or a recess of a large one, the amount of pus drawn through the needle may not be enough to show itself in the barrel of the syringe. I would recommend a careful examination of the contents of the needle after each puncture. At times a drop of pus will reward us, and another careful puncture in the same direction will strike the cavity. The number of punctures varies. I have made as many as six at one sitting. It is rather a painful procedure, and exhausts the patient seriously. It is also not free from danger. Bleeding from the liver often results, and I have seen quite an accumulation of blood in the abdominal cavity after diagnostic puncture. There is one case on record of fatal hemorrhage after aspiration.

It would probably be good practice to be ready to proceed with the operation as soon as aspiration revealed the presence of a pus collection, instead of waiting and running the risk of the leaking of pus into the peritoneal cavity. The character of the pus varies somewhat. It is usually described as chocolate colored, tomato soup colored, or the color of anchovy sauce. The consistence is usually very thick, but it varies much. In the fluid one can usually find portions of necrotic liver tissue (so-called liver stalactites). These, if floated out in water, are seen to consist of three or four liver lobules, with the branches of the portal vein, hepatic artery and bile ducts between them. Mixed with this one often finds flakes and masses consisting of true pus collections. Occasionally we find little or no tomato soup pus, but the whole collection consists of white, curdy, flaky material. Microscopically we find fatty degenerated liver cells in great quantities, numerous fatty granules, amorphous bodies, and even crystals, but very few true pus cells. If amœbæ are present they may show great motility. In cases that contain much curdy or white flaky matter, we usually find pyogenic or putrefactive organisms; and in some specimens bacteria and bacilli can be demonstrated.

The course of hepatic abscess, if left untouched, is usually unfavorable. Some cases are on record where the abscess has pointed externally or into some abdominal viscus (intestine or stomach) or into a bronchus, and recovery has followed; but these cases are very rare.

As a rule, long before this stage is reached, the liver has suffered so severely, and such a quantity of liver substance has been destroyed, that a fatal issue results from the altered metabolism of the body. Personally, I have never seen a case recover that opened spontaneously into a bronchus; although, by some writers, it is considered a favorable termination. I have seen two cases that came under my care coughing and expectorating typical hepatic pus, the result of the bursting of a liver abscess into the right lung. The patients were both very sick, and the expectoration varied in amount from time to time. Sometimes for forty-eight hours expectoration would cease, and the patient would complain of high fever and pain in the right hepatic region. This retention would then be followed by a profuse flow. One case I aspirated, but could not find the pus collection. Afterwards, at his earnest request, I attempted by operation to find the abscess cavity and drain from below. I failed signally to find it. The case lived for about six months and died from exhaustion. No autopsy was allowed. The second case I refused to explore, as aspiration failed to demonstrate a cavity. He lived for about eight months. Two other cases of similar nature have been under my care. They deserve a short description.

Case 1. W. W., aged 52. Had suffered from dysentery for almost two years. He came under my care in December, 1899, suffering from severe cough and expectorating great quantities of tomato soup material. This had been present for about two months and had occurred suddenly after a very prolonged and severe sickness, during which he had suffered from intense pain in the hepatic region with fever and great weakness. Examination of the chest revealed a bulging of the right lower costal zone, swelling and œdema of the parts, and in the axillary line covering the sixth, seventh, eighth, and ninth ribs, a large fluctuating abscess cavity. The liver was not apparently enlarged downwards. Dulness was present over the lower half of the right lung, and moist crepitant râles could be heard everywhere. A diagnosis was made of hepatic abscess, opening into a bronchus and penetrating the intercostal spaces.

*Operation.*—Under chloroform the subcutaneous abscess was evacuated. It contained chocolate-colored pus of a dreadful odor. Parts of two ribs were black and necrotic, and a hole passed between the ribs into the lower portion of the pleural cavity. This contained about a quart of stinking pus. It was evacuated, and the cavity washed out, and the soft tissues fastened around it with a purse-string suture. After this time the pus leaked alongside the tube. It was then thought advisable to remove the necrotic ribs and pack the large cavity with gauze. This was done, and a huge cavity was found large enough to hold two fists. An opening was seen through the diaphragm leading into a ragged cavity in the liver as large as an orange. A ragged hole led into the right lung, which was partly destroyed and formed part of the wall of the abscess cavity. It was one of the most horrible chasms I have ever seen. Each day it was carefully irrigated and packed with iodoform gauze. It held admost two yards of gauze. No pus was coughed up, but the patient was too exhausted to recover. As is often observed in these cases, no attempt at repair was seen, and he died in two weeks.

CASE 2. Max S., aged 42. Came under my care in September, 1899, with symptoms pointing to an abscess in the right lobe of the liver. He was suffering from the following symptoms: Remittent temperature; marked anæmia, blood examination showed slight leucocytosis, but no malarial organisms. Pain marked in the right lobe and at the point of the right shoulder. Liver enlarged considerably upwards but not downwards. He had been treated since July for malaria without result. Aspiration in the sixth right interspace in the anterior axillary line demonstrated typical hepatic pus at the depth of about three and one-half inches. Operation was performed under chloroform. About three inches of the sixth rib were removed without opening the pleural cavity. Then the pleura was incised. An immediate rush of air into the pleural cavity resulted, and the patient became deeply cyanosed. The diaphragm was seized with two pairs of catch forceps and lifted up to the wound, which it effectually plugged. It was then sutured with catgut to the edges of the opening in the parietal pleura, thus shutting off the pleural cavity completely. The diaphragm was then incised in the direction of the original wound. The liver was found adherent to the diaphragm. A rectal trochar was now passed into the liver and pus evacuated. Along the trochar a narrow-bladed knife was passed into the abscess cavity, along this a large director and along this a large sized glass tube sheathed with rubber tubing. The patient left the operating table in fair condition. For about two weeks syphon drainage was used and the patient's condition improved greatly. The temperature dropped almost to normal and his appetite greatly improved. Then the temperature became elevated again, appetite became capricious, and he suffered intensely from pain in the point of the right shoulder (a sign that is invariably present when drainage is poor). An anæsthetic was given again, and after some difficulty the tube was reinserted and drainage established again. Improvement occurred for a time, but soon the patient again became worse in spite of good drainage.

He remained in a stationary condition for about a month, pain being present almost all the time in the shoulder, sometimes so intense that opiates had to be given for its relief. A slight cough developed. Suddenly, without any warning, on December 3d, he coughed up about half a pint of hepatic pus. From this time on he gradually sank. In January signs of extreme malnutrition showed themselves. The wound failed to show any signs of healing. The soft tissues around became boggy and œdematous, then purple, circulation became sluggish, and eventually gangrene occurred.

In about three weeks' time portions of the fifth, sixth, and seventh ribs had become necrotic, and the intercostal structures had disappeared entirely. A cavity was exposed, the walls of which were formed by a ragged necrotic mass of lung, diaphragm and liver.

The odor was horrible. Expectoration of pus had almost ceased, but there was still a profuse discharge from the wound. The patient gradually died from exhaustion. No autopsy was allowed. There was no reason to believe that the abscess cavity was insufficiently drained after the second operation, but it is quite probable that a second abscess was present, which eventually penetrated the diaphragm and opened into a bronchus.

The results of cases submitted to operation is also a great lottery. I have many times seen death from exhaustion result even in well drained abscess cavities, when a large amount of liver substance has been destroyed by the disease. Just how much liver tissue is necessary to carry on the vital functions is not known. Occasionally, in very large abscess cavities, where apparently more than a quarter of the liver has been lost, recovery results; while in comparatively small ones, where the condition appears favorable, a fatal issue will follow. In all probability the favorable or unfavorable termination depends entirely on the amount of liver substance destroyed, and that, too, in cases otherwise unattended by septic complications. From this one can draw the logical conclusion that early evacuation of the abscess cavities is attended by the smallest mortality.

My own mortality has been 47 per cent. But some of the cases that recovered from the hepatic abscess succumbed within a year cr two to other diseases. I lost one from tuberculosis of the lungs, and another from a continuance of the original dysentery. All the death occurred from very large abscesses that had been neglected.

The course of some of these serious cases after the abscess has been evacuated is very curious. For a long period, sometimes a month, the patient seems to improve a little, or at the least to hold his own; his appetite is often enormous and he enjoys everything he eats. I have known a patient eat a dozen eggs a day, besides large quantities of chicken broth and tea, and their capacity for milk punches is positively astounding. The food is apparently well digested and the bowels move regularly and well, but the digested products are not further elaborated by the still healthy liver, and consequently the patient does not gain strength. After a time the patient begins to take the downhill course, bedsores appear, the wound become unhealthy, and death results.

*Treatment.*—There is only one treatment, and that is *evacuation.* This may be accomplished in the following ways: (1) Aspiration. (2) Tapping with a large trochar and cannula, followed by permanent drainage through the cannula. (3) Incision through the parietes and exposure of the liver, followed by evacuation of the abscess cavity.

*Aspiration.*—Nowadays, except for purpose of diagnosis, this is never employed. It is doubtful if a cure of a true hepatic abscess was ever obtained by this method.

2. *Permanent Drainage by Means of Cannula.*—This has been employed with success in many cases. It is, however, only safe in those abscesses that are more or less adherent to the parietes and where the peritoneal and pleural cavities are obliterated. After about forty-eight hours has elapsed, leaking occurs alongside the cannula, and there is danger of infection. I have found it of great value in draining deeply-seated abscesses after incision of the parietes, exposure of the liver and careful gauze packing around the proposed puncture. It is a very rapid and sure method, and if a large cannula is used (personally I use an Emmett's ovariotomy trochar and cannula), a piece of thick rubber tubing may be passed through it into the abscess cavity and the cannula immediately withdrawn, leaving the tubing in situ. In a few minutes the liver will contract on the tubing and drainage will be perfect without leaking, at least for forty-eight hours, when adhesions will have formed between the parietes and the abdominal contents.

One very ingenious way of using the cannula has been described by Manson. Through the cannula lying in the abscess cavity, a length of rubber drainage tube stretched longitudinally on a long director, is passed. When the end reaches the bottom of the abscess cavity the cannula is removed, leaving the tubing in the track of the cannula. Then the director is detached, care being taken to keep the deep end in contact with the deep wall of the abscess cavity. The rubber tubing immediately regains its original diameter and fills the tunnel through the liver substance, completely preventing any leaking of pus. The director is then withdrawn. (Lately Cantlie, *British Medical Journal*), February 22, 1902, has reported twenty-four recoveries out of twenty-eight cases operated on by this method.)

3. *Open Operation Through the Parietes.*—These operations are best described according to whether the incision is through the abdominal or thoracic walls.

(*a*) *Through the Abdominal Wall.*—The incision through the parietes is made over the position of the pus collection in the case of abscesses in the right lobe. In those occupying the left lobe it had best be made in the median line or through the fibres of the left rectus muscle. If the liver is adherent the operation resolves itself in a simple opening of an abscess cavity. If no adhesions exist we can proceed in one or two ways. First. Surround the point of puncture with gauze packing and evacuate the pus immediately with trochar and cannula or simple incision, as seems best; or, secondly, secure adhesions before evacuation by careful suture of the liver to the parietal peritoneum. This is rather difficult to accomplish, and unless done carefully the respiratory movements will tear the stitches out of the friable liver substance. It can be accomplished in the following way: Carefully separate the peritoneum from the parietes around the incision, until you have a curtain of movable membrane two inches long. Then carefully elevate a similar ring flap from the liver around the site of the proposed puncture (this is impossible in some cases, but in others I have easily accomplished it). Then suture the parietal and visceral flaps of peritoneum together, thus shutting off the peritoneal cavity completely. The object of the flap is to allow for the respiratory movements. After adhesions have been secured the evacuation of the abscess cavity may be immediately proceeded with, and the wound may be packed for forty-eight hours before evacuation of the abscess.

When the flaps are long enough there is absolutely no danger of tearing. In one case of very large abscess in the right lobe, where I succeeded in uniting liver and parietal peritoneum very successfully, I drained immediately with a large cannula. The following day the patient withdrew the cannula and walked about his bedroom. I replaced it again with some difficulty, and fully expected serious consequences, but the case proceeded to recovery without an unfavorable symptom.

(*b*) *Through the Thoracic Wall.*—In conducting operations by this route the most important point is to determine the level of the lower edge of the lung and the condition of the lower part of the pleural cavity. If the liver dullness is much elevated, the chances are that the lung is pushed upwards, and that its lower edge does not descend into the lower part of the pleural cavity. By turning the patient on his left side the upper level of the liver dullness will always descend a little (unless adhesions are present), and if in this position we find in deep inspiration that the lower part of the chest wall is not at all resonant, we may assume that the diaphragm and parietes are in contact at this spot. The best plan to pursue is to assume that no adhesions are present. The best place to operate is at the lower part of the chest in the mid-axillary line. This spot, however, can not always be used because aspiration here will not always demonstrate pus. It should, however, be a rule to aspirate first as near as possible to the lower reflection of the pleura rather than higher up, because operation being always conducted along the track of aspirating needle, fewer difficulties are encountered in this locality.

Practically the only dangers in the trans-pleural operation are the following: (1) Entrance of air into the pleural cavity and collapse of the lung. (2) Septic infection of the pleural cavity. (3) Septic infection of the peritoneal cavity. We can avoid these by the following technique: An incision from three to four inches long is made along one of the ribs nearest to the aspiration puncture that found pus. The rib is exposed and a length is resected subperiostially, care being taken not to wound the parietal pleura. As soon as the bleeding has been stopped the parietal pleura is incised and the diaphragm exposed. If perchance (as in one of cases described previously) air enters the pleural cavity, the diaphragm should be seized with forceps and lifted up into the wound as a plug. The margins of the parietal pleura are now carefully stitched to the diaphragm, so shutting off the pleural cavity. If, as is usually the case, the liver is considerably enlarged upwards and the costal and diaphragmatic levers of the pleura are in contact, no air will enter into the pleural cavity. (I have only seen it do so once in the case above quoted.) Then an incision should be made into the diaphragm parallel to the original wound and the diaphragm split, a flap being thrown to either side. The edges of the diaphragmatic flaps should be united to those of the costal pleura.

Then the rest of the diaphragm is divided and the pritoneal cavity opened. The liver now comes into view, and on its surface the puncture of the aspirating needle will be seen. A flap of peritoneum is now raised up from the liver. This will necessitate the inclusion of some superficial liver tissue in the flap. This part of the operation is often tedious and attended with a fair amount of oozing. At times it is impracticable, on account of the friability of the liver tissue or the close proximity of the pus, in which event gauze packing should be carefully placed around the site of the proposed puncture between the upper surface of the liver and diaphragm. The evacuation of the pus should be conducted as previously described, preferably by a large trochar and cannula, the cannula being withdrawn over a large rubber tube which is left in situ.

This operation is ideal and fulfills every indication. Whether Manson's operation is superior can not yet be settled. In very extreme cases in which it is inadvisible to submit the patient to a moderately prolonged anæsthesia, it would probably be better to employ Manson's method, but in the others I feel sure that this method is much superior.

Of course, there are some cases where the pus is so near the surface that the pleural cavity is obliterated, the diaphragm is unrecognizable and the liver is adherent to the diaphragm. Here the operation is easy, and is simply a dissection until liver substance is recognized when the abscess is drained.

In abscesses occupying the left lobe of the liver, to secure adhesions before evacuation of the cavity is impossible. Here we are forced to use a gauze dam inside which the abscess is evacuated. In these cases slow evacuation is always desirable to prevent undue soiling of the gauze. By the use of the large trochar and cannula this can be accomplished perfectly.

*The after treatment* requires great care. The tube becomes blocked up constantly with plugs of degenerated liver tissue. The best way of securing good drainage is by Cathcart's hydrostatic pump, using a Y glass tube instead of a T. The exit tube can often be emptied by milking it. At times a plug of liver tissue can not pass, and the tube remains blocked for a few hours. By reversing the milking process the plug can often be forced back into the abscess cavity, allowing the thinner material to flow again.

Another troublesome feature is the tendency of the liver to shrink considerably. This shrinkage pulls the wound in the liver away from that in the parietes, and unless great care is taken daily to push the tube to the bottom of the abscess cavity the tube may gradually slide out of it. This accident occurs much more frequently in downward than in upward enlargement of the liver. It requires immediate rectification, otherwise the track may heal up and another operation become necessary. Flushing of the cavities is rarely necessary. In fact, it is probable that it conduces to the very danger we wish to avoid, viz., infection of the abscess cavity with pyogenic organisms before the walls are able to resist the infection. I would make one exception to this. In abscess cavities that contain white flaky pus and but little chocolate-colored material, the walls of the abscess cavity consist of hard tuberculated chronically inflamed liver tissue. These cavities can be completely evacuated through a large opening and the walls cleaned, after which the cavity can be packed with iodoform gauze. My experience has been that these cases are very favorable, and a speedy recovery occurs, whereas, in the abscess containing chocolate-colored material exclusively, the walls consist of soft friable velvety slimy material consisting of necrotic liver tissue, which gradually passes into healthy liver. No amount of cleaning will take this away at the time of operation. It comes away gradually along the drainage tube, being thrown off as sloughs are separated in other parts of the body.

The exact thickness of this necrotic layer can seldom be estimated. I have seen one liver at an autopsy where it was fully two inches thick.

Sometimes the necrotic process does not terminate with the evacuation of the cavity. In these cases the bad symptoms continue and the patients slowly succumb.

The possibility of a second abscess must always be considered. Where, in spite of good drainage of the original abscess, bad symptoms continue, such a condition is probable, and further aspiration should be resorted to. Such a case was under my care in April, 1903. A negro was admitted to the hospital with a large abscess in the right lobe of the liver. The liver was enormously enlarged in a downward direction. The lower edge reached fully a hand's breadth below the rib margin. It moved freely with respiration, and there was no friction sound to be heard. There was a clear history of dysentery and amœbæ in large quantities were discovered in the stools. The temperature was remittent. The highest point was reached in the evening and varied between 103° and 104°. The lowest was in the morning and rarely fell lower than 100°. He was greatly emaciated and complained of severe pain over the liver.

Over the most prominent point of the swelling in the semilunar line, about one and one-half inches below the costal margin, a sensation of fluctation was elicited.

An incision was made through the abdominal wall at the outer edge of the rectus muscle, and the peritoneal cavity opened. A roughened tuberculated liver presented. A peritoneal flap was dissected from the parietes, but an attempt to dissect one from the liver failed. Gauze packing was placed in position and the abscess cavity evacuated through a large cannula. Rubber tubing was passed along the cannula which was then withdrawn. The abscess was a very large one, and contained fully a quart of chocolate-colored pus, which was full of very active amœbæ. Otherwise it was sterile.

The patient progressed very favorably for about two weeks. Then the temperature began to rise and the pain to return. Examination showed that the shrinkage of the liver had dragged it away from the tube, which no longer lay in the abscess cavity. Under anæsthesia it was replaced and the examining finger found that the abscess cavity was replaced to one-fifth of its original size. Still the fever rose. Two weeks later a second large abscess was found by the side of the first, and apparently not connected with it. The contents were evacuated and the patient's condition began to improve. He is still under treatment at time of writing.

